# Implicit Associations and Explicit Expectancies toward Cannabis in Heavy Cannabis Users and Controls

**DOI:** 10.3389/fpsyt.2013.00059

**Published:** 2013-06-21

**Authors:** Esther M. Beraha, Janna Cousijn, Elisa Hermanides, Anna E. Goudriaan, Reinout W. Wiers

**Affiliations:** ^1^ADAPT-Lab, Department of Psychology, University of Amsterdam, Amsterdam, Netherlands; ^2^Amsterdam Institute for Addiction Research, Academic Medical Center, Department of Psychiatry, University of Amsterdam, Amsterdam, Netherlands; ^3^Brain and Development Lab, Department of Psychology, Leiden University, Leiden, Netherlands

**Keywords:** cannabis, implicit association test, cannabis use disorder, craving, affective associations

## Abstract

Cognitive biases, including implicit memory associations are thought to play an important role in the development of addictive behaviors. The aim of the present study was to investigate implicit affective memory associations in heavy cannabis users. Implicit positive-arousal, sedation, and negative associations toward cannabis were measured with three Single Category Implicit Association Tests (SC-IAT’s) and compared between 59 heavy cannabis users and 89 controls. Moreover, we investigated the relationship between these implicit affective associations and explicit expectancies, subjective craving, cannabis use, and cannabis related problems. Results show that heavy cannabis users had stronger implicit positive-arousal associations but weaker implicit negative associations toward cannabis compared to controls. Moreover, heavy cannabis users had stronger sedation but weaker negative explicit expectancies toward cannabis compared to controls. Within heavy cannabis users, more cannabis use was associated with stronger implicit negative associations whereas more cannabis use related problems was associated with stronger explicit negative expectancies, decreasing the overall difference on negative associations between cannabis users and controls. No other associations were observed between implicit associations, explicit expectancies, measures of cannabis use, cannabis use related problems, or subjective craving. These findings indicate that, in contrast to other substances of abuse like alcohol and tobacco, the relationship between implicit associations and cannabis use appears to be weak in heavy cannabis users.

## Introduction

Cannabis is the most widely used illegal substance in most countries and treatment demands for cannabis dependence directly follow demands for alcohol and opiates (Degenhardt et al., 2008; UNODC, 2009). However, relatively little is known about the processes underlying continued cannabis use and the eventual progression toward dependence. Theoretical models of addiction suggest that relatively automatically triggered motivations to use play an important role in the development and persistence of addictive behaviors (Wiers et al., 2007a; Koob and Volkow, 2010). A better understanding of the motivational processes underlying cannabis use may therefore help to gain more insight into cannabis abuse and dependence.

In regular substance users, substance use has repeatedly been paired with certain cues, such as paraphernalia, specific contexts, or emotional states. This is thought to cause the substance users’ brain to become extremely sensitive toward these cues. Subsequent exposure to substance-related cues may then relatively automatically bias motivation toward substance use. Indeed, prior studies showed that substance-related cues automatically capture attention (i.e., attentional bias; e.g., Field, 2005; Mogg et al., 2005), elicit approach tendencies (i.e., approach bias; e.g., Field, 2005; Mogg et al., 2005; Wiers et al., 2009), and activate implicit memory associations in heavy substance users (e.g., Wiers et al., 2002; Field et al., 2004). These so called “cognitive biases” are thought to be relatively automatically triggered, the may reach awareness (for reviews, see McCusker, 2001; Wiers et al., 2007a). Furthermore, cognitive biases tend to correlate with subjective craving (e.g., Franken et al., 2000; Mogg et al., 2003; Field et al., 2004; Waters et al., 2007), although not consistently over studies (Field et al., 2009). Additionally, cognitive biases have been found to predict relapse in cigarette smokers (Waters et al., 2003; Kahler et al., 2007), heroin dependent individuals (Marissen et al., 2006; Marhe et al., 2013), and alcohol dependent individuals (Cox et al., 2002).

Indirect or implicit measures of drug-related motivations like the aforementioned cognitive biases are considered promising in the field of addiction since they are less prone to self-awareness (De Houwer et al., 2009). This is especially important as substance-related cues have been shown to activate motivational brain circuits outside conscious awareness (Childress et al., 2008) and substance users may lack insight in cognitive processes underlying their own behavior (Goldstein et al., 2009).

One test that has often been used to assess implicit memory associations is the Implicit Association Test (IAT; Greenwald et al., 1998). The underlying idea of this task is that the categorization of associated stimuli (e.g., flowers and positive words) is easier compared to the categorization of non-associated stimuli (e.g., flowers and negative words). The IAT has extensively been used to assess implicit memory associations toward substances of abuse, such as alcohol and cigarets (for meta-analyses, see Rooke et al., 2008; Reich et al., 2010; for review, see Roefs et al., 2011). Previous IAT studies demonstrated that both light drinkers and heavy drinkers had implicit negative associations toward alcohol (e.g., Wiers et al., 2002; De Houwer et al., 2004). Interestingly, it was found that heavy drinkers do have stronger positive alcohol-arousal compared to negative alcohol-sedation associations, whereas light drinkers don’t show this effect (Wiers et al., 2002). Furthermore, implicit associations were found to predict alcohol use (Ostafin et al., 2008). Similarly to heavy alcohol users, smokers tend to have implicit negative associations toward smoking, however, often less strong than non-smokers (Roefs et al., 2011). Furthermore, implicit associations in smokers have been found to correlate with self-reported smoking, relapse, and craving (Roefs et al., 2011). In contrast to heavy alcohol users and smokers, cocaine dependent patients do not show implicit negative associations toward cocaine, but do show stronger arousal, positive, and sedation associations compared to controls (Wiers et al., 2007b). Moreover, a recent study in heroin dependent patients showed that implicit positive association could predict relapse (Marhe et al., 2013).

To date, studies investigating implicit memory associations toward cannabis are sparse. In a study by Field et al. (2004) positive and negative implicit associations for cannabis related words were examined in monthly to daily cannabis users and controls. It was found that cannabis users showed weaker implicit negative associations for cannabis compared to controls, whereas the implicit positive associations did not differ between groups. Furthermore, no correlations were found between implicit associations and craving or other measures of cannabis use. In a second study implicit and explicit, positive-arousal, sedation, and negative associations were examined in a heterogeneous group of adolescent cannabis users (cannabis use ranged from never to daily, Ames et al., 2007). Using Single Category Implicit Association Tests (SC-IATs; Karpinski and Steinman, 2006), it was found that implicit positive-arousal associations toward cannabis predicted cannabis use, over and beyond explicit measures of affective expectancies. Moreover, explicit sedation associations also strongly predicted cannabis use. Finally, a study using similar SC-IATs compared implicit positive-arousal, sedation, and negative associations toward cannabis between male patients with schizophrenia and healthy controls (cannabis use ranged from never to daily in both groups, Dekker et al., 2010). Implicit associations did not differ between groups, however, the patients showed stronger explicit negative expectancies toward cannabis compared to controls. Moreover, explicit sedation associations were positively associated with craving and cannabis use.

These inconsistent findings and methodological differences (e.g., quantitative cannabis use, unipolar SC-IATs vs. bipolar IAT’s, and sample characteristics) between studies preclude drawing strong conclusions about the relationship between implicit memory associations and heavy cannabis use. The primary aim of the present study therefore was to investigate implicit affective memory associations in a large sample of non-treatment seeking heavy cannabis users compared to controls. Moreover, within the group of heavy cannabis users we assessed the relationship between these implicit affective associations and explicit expectancies, craving, quantitative cannabis use, and severity of cannabis use related problems. Similar to Ames et al. (2007) and Dekker et al. (2010), participants performed three SC-IATs to measure implicit associations in three dimensions: positive-arousal (excitement), negative (negative affect), and sedation (negative reinforcement) associations toward cannabis use. We hypothesized that cannabis users would show stronger implicit arousal associations (Ames et al., 2007), explicit sedation cannabis associations correlating with cannabis use and craving (Ames et al., 2007; Dekker et al., 2010), whereas controls would have stronger implicit negative associations (Field et al., 2004). Furthermore, we expected to find positive correlations between negative implicit associations and cannabis related problems.

## Materials and Methods

### Participants

For the current study data from two separate studies investigating neurocognitive processes related to cannabis use were combined (see also Cousijn et al., 2011, 2012, 2013). From these studies participants that completed the IATs were included, resulting in a sample of 59 heavy cannabis users and 89 controls aged 18–25. The first study included 26 heavy cannabis users (male/female = 15/11) and 47 controls (male/female = 7/40) and was conducted at the testing facilities of the Psychology faculty of the University of Amsterdam. The second study was a neuroimaging study conducted at the Academic Medical Center of the University of Amsterdam including 33 heavy cannabis users (male/female = 21/12) and 42 controls (male/female = 27/15). In this study the IATs were performed outside the scanner near the end of the test session. Participants were recruited through advertisements on the internet and in cannabis outlets (coffee-shops). Groups were matched for age and estimated intelligence (Schmand et al., 1991), see Table 1. Heavy cannabis use was defined as using cannabis for at least 10 days in the previous month, for 240 or more days in the past 2 years, and not seeking treatment or having a history of treatment for cannabis use. Participants in the control group used cannabis on fewer than 50 life-time occasions and did not use in the previous year. To control for other substance and alcohol use, participants with an Alcohol Use Disorder Identification Test (AUDIT; Saunders et al., 1993) score higher than 10, who smoked more than 20 cigarets per day, and who used any non-cannabinoid drugs on more than 100 occasions during life (all participants<25 occasions) were excluded. Further exclusion criteria were a history of major medical, physical, or psychiatric disorders, assessed with the Mini-International Neuropsychiatric Interview (M.I.N.I., Sheehan et al., 1998; Dutch version 5.0.0). The study was approved by the Medical Ethics Committee of the Academic Medical Centre and the Ethics Committee of the University of Amsterdam and all participants signed informed consent before participation.

**Table 1:** **Sample characteristics**.

	Heavy cannabis users	Controls
N (% female)	59 (39)	89 (62)*
Age, mean (SD)	21.3 (2.9)	21.6 (3.2)
Verbal IQ (Dutch Reading Test), mean (SD)	105.6 (5.7)	106.4 (6.2)
Alcohol use and related problems (AUDIT), mean (SD)	7.8 (4.9)	7.0 (4.0)
Cigarette smoking (%)	70	32**
Nicotine dependence (FTND), mean (SD)	2.7 (2.2)	1.2 (1.9)**
Cannabis use and related problems (CUDIT), mean (SD)	12.0 (6.0)	0.5 (1.0)**
Duration heavy cannabis use (year), mean (SD)	2.2 (1.8)	–
Current cannabis use days/week, mean (SD)	4.2 (1.9)	–
Current cannabis use gram/week, mean (SD)	2.6 (2.3)	–
**CRAVING**		
MCQ compulsivity	7.6 (4.3)	3.3 (1.3)**
MCQ emotionality	7.8 (4.1)	3.8 (1.5)**
MCQ expectancy	8.9 (3.5)	4.0 (1.9)**
MCQ purposefulness	13.0 (6.0)	5.0 (2.7)**

*p < 0.05 and **p < 0.001 for group comparison. SD, standard deviation; AUDIT, alcohol use disorder identification test; FTND, Fagerström Test for Nicotine Dependence; CUDIT, cannabis use disorder identification test; MCQ, Marijuana Craving Questionnaire.

### Questionnaires

The Cannabis Use Disorder Identification Test (CUDIT; Adamson and Sellman, 2003) was used to assess cannabis use and related problems. The CUDIT is a screening instrument for at-risk cannabis use and consists of 10 items on cannabis use frequency, symptoms of dependence, and use related problems (Adamson and Sellman, 2003; Adamson et al., 2010). Furthermore, detailed information about past and present cannabis use was obtained, such as duration of use, weekly use (days, grams), and life-time use. Tobacco use and dependence was measured with the Fagerstrom Test for Nicotine Dependence (FTND; Heatherton et al., 1991).

The short 12-item version of the Marijuana Craving Questionnaire (MCQ; Heishman et al., 2009) was used to assess subjective craving after the test session. The MCQ is reliable for assessing craving in non-treatment seeking cannabis users (Heishman et al., 2001, 2009). The MCQ distinguishes four three-item craving factors: compulsivity (inability to control use, e.g., “I need to smoke marijuana now”), emotionality (relief from withdrawal and negative affect, e.g., “I would feel less anxious if I smoked marijuana right now”), expectancy (anticipation of positive outcomes, e.g., “smoking marijuana would make me content”), and purposefulness (planning/intention to use for positive outcomes, e.g., “smoking marijuana would be pleasant right now”). Items were rated on a Likert response scale ranging from 1 (strongly disagree) to 7 (strongly agree).

### Implicit association test

Participants completed three SC-IATs in a row to assess implicit affective associations toward cannabis. The SC-IAT’s were presented on a computer screen with E-prime software (version 2.0, Psychology Software Tools, Inc.). Each SC-IAT measured one of the three different affective associations toward the use of cannabis: positive-arousal, sedation, and negative affect. Each affective category was compared to a neutral category. The SC-IAT’s were performed in random order, counterbalanced over participants and groups. Each SC-IAT contained four categories; two target categories (“cannabis” or “other”) and two attribute categories (i.e., “excited” or “neutral”). Stimuli in the target categories were five cannabis related pictures (i.e., joint, weed) and five neutral pictures (stationeries), matched on color and image composition. Stimuli in the attribute categories were five affective and five neutral words. These words were matched on the number of letters, syllables, familiarity, valence, and arousal.

Each SC-IAT consisted of five blocks. The first block was a target discrimination practice block (e.g., left = cannabis and right = other), consisting of 20 trials, in which each image was presented two times. Participants were asked to categorize the images to one of the target categories by pressing a corresponding button (e-key = left or i-key = right). The second block was an attribute discrimination practice block consisting of 20 trials in which participants had to categorize words to one of the attribute categories. The third block was a combination block consisting of 20 practice and 20 test trials, in which target and attribute categories were presented together (left = cannabis + negative and right = other + neutral). Participants were now required to categorize stimuli to a target category combined with an attribute category. After this combination block, the target categories were reversed and practiced in another target discrimination practice block (e.g., left = other and right = cannabis). The final block was a second combined categorization block of 20 practice and 20 test trials (e.g., left = cannabis + neutral and right = other + negative). The order of the SC-IAT’s and the combination blocks were counterbalanced. The IAT effect was considered the difference in RT between the two combined categorization blocks. Thus, participants with an implicit negative cannabis association were faster in responding to the cannabis-negative combined blocks compared to the cannabis-neutral combined blocks.

Each trial started with a word or image presented in the center of the screen. Target and attribute words were presented on the left and right top of the screen in order to remind participants of the categories. Participants were instructed to categorize stimuli as quickly as possible by pressing a left or right response button with their index fingers. If an incorrect response was made, participants saw a red “X” on the screen and were asked to correct their response before the next trial started. If participants did not response within 2500 ms, they received the feedback “too slow.”

### Explicit expectancies

Similarly to Ames et al. (2007) and Dekker et al. (2010), explicit cannabis expectancies were assessed with 29 items consisting of equivalent words used in the three SC-IATs. Statements on cannabis use and positive-arousal (i.e., if I smoke cannabis I feel excited), negative (i.e., I feel sick), and sedation (i.e., I feel relaxed) expectancies had to be rated. Participants were instructed to indicate the extent to which they agreed or disagreed with the statements on a Likert response scale ranging from 1 (strongly disagree) to 6 (strongly agree). Internal reliability of the scales in the presents sample was good: positive-arousal expectancies Cronbach’s α = 0.86, negative expectancies Cronbach’s α = 0.93, and sedation expectancies Cronbach’s α = 0.95.

### Procedure

Test sessions took place during the late afternoon and at the beginning of the evening. All participants were asked to refrain from alcohol and drug use 24 h prior to testing (this was verified with urine analysis in study 2). Each session started with signing the informed consent form. After completing questionnaires and the diagnostic interview, participants performed the three SC-IATs. Participants were then asked to rate the statements on cannabis use expectancies. Following Field et al. (2004), craving was assessed at the end of the test session.

### Statistical analysis

Participants who made more than 35% errors during the IATs and with an average RT more than three standard deviations (SD) above or below the group mean were considered outliers and omitted from the analyses. The D2SD measure is considered the standard approach when analyzing the IAT (Greenwald et al., 2003). For each of the three affective dimensions a D2SD measure was therefore calculated based on the scoring algorithm provided by Greenwald et al. (2003). The D2SD measure is calculated from the RT difference between different affective categorizations blocks (e.g., cannabis-negative vs. cannabis-neutral), corrected for the standard deviation of these blocks to minimize the influence of RT variance between participants. Correcting for the standard deviation is especially important when comparing groups in which RT variability may differ between groups. Both the practice and test trials of a combination block are included in the analysis. Moreover, an error penalty is used by replacing the RT of each error trial with the mean RT plus twice the standard deviation of all correct trial of the block. To investigate main effects of the Dependent Variables group, study^1^, and gender, a multivariate analysis of variance (MANOVA) was performed. A MANOVA assumes a linear relationship between any two Dependent Variables. We performed separate MANOVAs for the implicit memory associations and the explicit expectancies because we cannot assume a linear relationship between implicit memory associations and the explicit expectancies. Moreover, since it cannot be assumed that the implicit measures are deviations of one dependent variable, a multivariate approach was chosen over a univariate repeated measures ANOVA. To further investigate differences between heavy cannabis users and controls discriminant analyses were conducted (Huberty and Morris, 1989), taking into account the relationship between the dependent variables (the three implicit memory associations or the three explicit expectancies). One-sample *t*-tests were used to test if IAT scores differed significantly from zero within each group. In order to investigate associations between implicit associations and explicit expectancies, craving, cannabis use, and cannabis use related problems Pearson’s correlations were calculated. To control for the potential confounding effects of nicotine use, within the group of heavy cannabis users IAT scores were correlated with scores for nicotine dependence and smokers and non-smokers were compared.

## Results

### Sample characteristics

The IAT scores of three controls were excluded from analysis because their errors exceeded 35% (40–49%). Furthermore, one control was discarded as outlier because the IAT score was above four SD from the group mean (results were similar when this participant was included in the analyses). The groups did not differ in age (*t*_148_ = 0.57, *p* = 0.57), IQ (*t*_148_ = 0.79, *p* = 0.43), and alcohol use (*t*_148_ = 1.03, *p* = 0.31). However there were more men (*x*^2^ = 7.41, *p* = 0.006) and more cigarette smokers (*x*^2^ = 20.62, *p* < 0.001) in the heavy cannabis users group. CUDIT scores (*t*_148_ = 17.73, *p* < 0.001) and craving (compulsivity: *t*_148_ = 8.81, *p* < 0.001, emotionality *t*_148_ = 8.37, *p* < 0.001, expectancy *t*_146_ = 10.84, *p* < 0.001, and purposefulness *t*_148_ = 11.07, *p* < 0.001) were higher in the heavy cannabis users compared to controls (see Table 1).

### Implicit and explicit memory associations

#### Group comparison

A MANOVA was performed to investigate main effects of group, study, and gender on implicit memory associations. There was a significant main effect of group (*F*_3, 135_ = 4.76, *p* = 0.003, η^2^ = 0.10), but not for study (*F*_3, 135_ = 0.89, *p* = 0.45) or gender (*F*_3, 135_ = 2.13, *p* = 0.10). No significant interactions between group and study (*F*_3, 135_ = 0.71, *p* = 0.55), or group, and gender (*F*_3, 135_ = 0.45, *p* = 0.72) were found.

**Figure 1:**
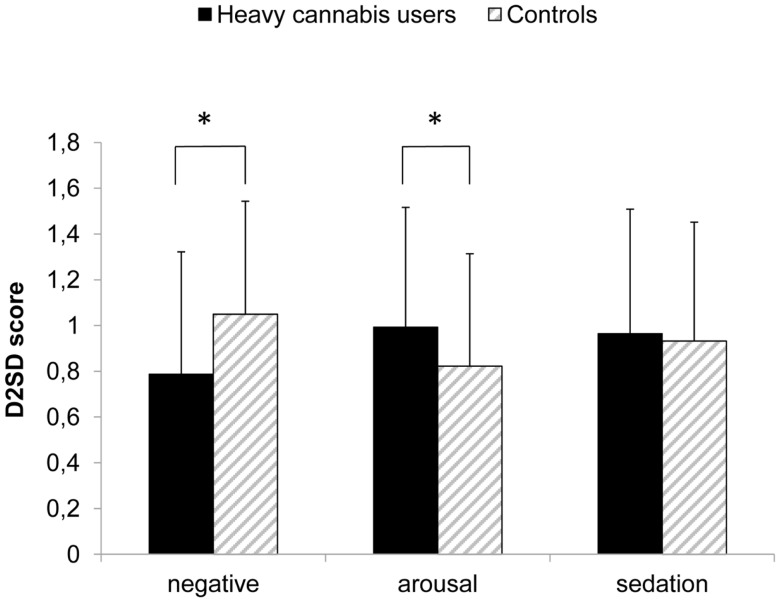
**Implicit associations (IAT D2SD score) for each of the three affective IAT dimensions**. Means + standard deviations are depicted per group. *p < 0.05.

A discriminant analysis, which focused on the structure coefficients, was performed to determine the relative contribution of the different implicit memory associations to the main effect of group (Huberty and Morris, 1989). The relative contributions to the difference between heavy cannabis users and controls were: implicit negative associations (−0.93), positive-arousal associations (0.64), and sedation associations (0.30). Groups differed significantly in negative (*t*_144_ = 3.04, *p* < 0.01) and positive-arousal associations (*t*_144_ = 2.01, *p* = 0.046), with heavy cannabis users showing weaker negative associations (mean D score = 0.79, SD = 0.53) compared to controls (mean D score = 1.05, SD = 0.49) and stronger positive-arousal associations toward cannabis (mean D score = 0.99, SD = 0.52) compared to controls (mean D score = 0.82, SD = 0.50). These effects are illustrated in Figure 1. *Post hoc* one-sample *t*-tests indicated that both groups had a significant negative, arousal, and sedation association toward cannabis (*t* < 11.30, *p* < 0.001).

**Figure 2:**
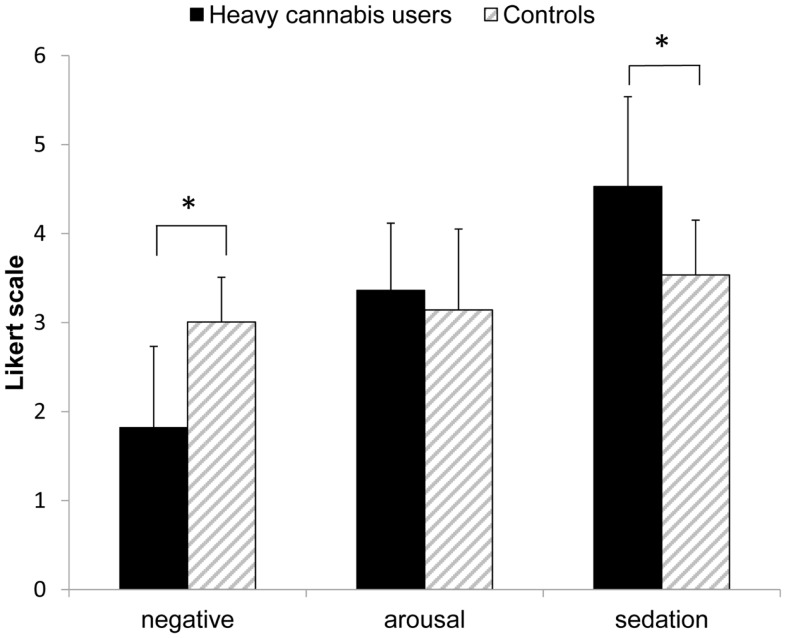
**Explicit expectancies (Likert scale) for each of the three affective IAT dimensions**. Means + standard deviations are depicted per group. *p < 0.001.

With regard to the explicit expectancies, a MANOVA revealed main effects for group (*F*_3, 138_ = 21.25, *p* < 0.001, η^2^ = 0.32) and study (*F*_3, 138_ = 4.66, *p* = 0.004, η^2^ = 0.09) but not for gender (*F*_3, 138_ = 1.76, *p* = 0.16). A discriminant analysis showed that the relative contributions to the difference in explicit expectations between groups were: negative expectancies (0.81), sedation expectancies (−0.36), and positive-arousal expectancies (0.22). As shown in Figure 2, differences between groups were found in negative (*t*_146_ = 9.10, *p* < 0.001) and sedation expectancies (*t*_146_ = 6.78, *p* < 0.001), with controls showing stronger negative expectancies (mean D score = 3.01, SD = 0.91) compared to cannabis users (mean D score = 1.82, SD = 0.50) and heavy cannabis users showing stronger relaxed expectancies (mean D score = 4.53, SD = 0.62) compared to controls (mean D score = 3.54, SD = 1.01). No significant differences between groups were found in positive-arousal expectancies (*t*_146_ = 1.56, *p* = 0.11). Participants differed between studies concerning positive-arousal expectancies (*t*_146_ = 4.00, *p* < 0.001), with the participants tested in the Academic Medical Center showing weaker positive-arousal expectancies compared to those tested at the Psychology faculty.

#### Correlations

In the heavy cannabis users group, no significant correlations between implicit and explicit associations were found. See Table 2 for a full overview of the correlations between implicit associations, explicit expectancies, cannabis use, and craving within the heavy users group. In the control group implicit positive-arousal associations and explicit positive-arousal expectancies (*r* = 0.23, *p* = 0.033) and implicit relaxed associations and explicit relaxed expectancies (*r* = 0.26, *p* = 0.012) correlated positively. There was no significant correlation between implicit and explicit negative associations (*r* = −0.01, *p* = 0.958). See Table 3 for a full overview of the correlations between implicit associations and explicit expectancies.

**Table 2:** **Correlation matrix within heavy cannabis users: implicit associations, explicit expectancies, cannabis use, and craving**.

		Implicit associations (IAT)			Explicit expectancies			Cannabis use and problems				Craving (MCQ)			
		Negative	Positive-arousal	Sedation	Negative	Positive-arousal	Sedation	Cudit	Gram per week	Days per week	Duration use	Compulsivity	Emotionality	Expectancy	Purposefulness
Implicit associations (IAT)	Negative	1.00													
	Positive-arousal	0.14	1.00												
	Sedation	0.23	0.16	1.00											
Explicit expectancies	Negative	−0.12	0.11	−0.07	1.00										
	Positive-arousal	0.03	0.14	0.04	−0.13	1.00									
	Sedation	0.22	0.15	0.21	−0.54**	0.35*	1.00								
Cannabis use and problems	Cudit	0.12	0.11	−0.06	0.29*	−0.09	−0.17	1.00							
	Gram per week	0.28*	0.01	−0.02	−0.15	0.07	0.02	0.48**	1.00						
	Days per week	0.02	0.07	−0.20	0.01	−0.04	0.00	0.42**	0.69**	1.00					
	Duration use	0.06	0.06	−0.04	−0.04	−0.01	−0.18	0.03	0.13	0.18	1.00				
Craving (MCQ)	Compulsivity	−0.03	0.00	0.10	0.15	0.06	0.06	0.58**	0.45**	0.39*	−0.08	1.00			
	Emotionality	−0.05	0.12	−0.06	0.16	0.22	0.18	0.35*	0.14	0.07	−0.12	0.68**	1.00		
	Expectancy	0.09	0.01	0.02	0.06	0.07	0.07	0.18	0.35*	0.34*	0.08	0.53**	0.46**	1.00	
	Purposefulness	0.00	−0.06	0.03	0.00	0.22	0.21	0.37*	0.50**	0.43**	−0.07	0.81**	0.60**	0.54**	1.00

*p < 0.05, **p < 0.001, MCQ, Marijuana Craving Questionnaire; IAT, implicit association test.

**Table 3:** **Correlation matrix within controls: implicit associations and explicit expectancies**.

		Implicit associations (IAT)			Explicit expectancies		
		Negative	Positive-arousal	Sedation	Negative	Positive-arousal	Sedation
Implicit associations (IAT)	Negative	1.00					
	Positive-arousal	0.30*	1.00				
	Sedation	0.38**	0.26*	1.00			
Explicit expectancies	Negative	−0.01	−0.11	−0.13	1.00		
	Positive-arousal	0.27*	0.23*	0.23*	−0.34**	1.00	
	Sedation	0.24*	0.12	0.26*	−0.59**	0.62**	1.00

*p < 0.05, **p < 0.001, IAT, implicit association test.

Within the group of heavy cannabis users, implicit negative associations correlated positively with weekly cannabis use (grams, *r* = 0.28, *p* = 0.038), indicating that more cannabis use was associated with stronger implicit negative associations. No other significant correlations were found between implicit measures and measures of cannabis use, cannabis use related problems, or craving.

Within the group of heavy cannabis users, a positive correlation between CUDIT scores and explicit negative expectancies was found (*r* = 0.29, *p* = 0.026), indicating that cannabis users with more cannabis related problems had stronger explicit negative expectancies toward cannabis.

## Discussion

The present study aimed at investigating affective implicit memory associations toward cannabis in heavy cannabis users and controls. These implicit affective memory associations are considered to be fast and relatively automatically triggered (and not necessarily unconscious). Significant group differences in the IATs measuring implicit negative and positive-arousal associations were found: heavy cannabis users had stronger positive-arousal but weaker negative associations toward cannabis compared to controls. Positive-sedation associations did not differ between groups. Within the group of heavy cannabis users, weekly cannabis use (grams) was associated with stronger implicit negative associations, decreasing the difference between heavy cannabis users and controls. Regarding explicit affective expectancies, heavy cannabis users had stronger sedation but weaker negative expectancies toward cannabis compared to controls. In contrast to our hypothesis, we did not observe any other association between implicit memory associations, explicit expectancies, and measures of cannabis use, problems, or craving.

Both cannabis users and controls had significant implicit negative associations toward cannabis, although heavy cannabis users had weaker negative associations compared to controls. This negative association toward cannabis in heavy cannabis users is in accordance with Dekker et al. (2010) who, using a similar SC-IAT, reported implicit negative associations toward cannabis in both patients with schizophrenia and controls with sporadic to heavy levels of cannabis use. However, in the study of Field et al. (2004) negative associations toward cannabis were only observed in controls, not in heavy cannabis users. The latter finding may be explained by the use of a bipolar IAT by Field et al. (2004), in which negative associations were directly compared with positive associations (Karpinski and Steinman, 2006). Nevertheless, bipolar IAT studies in heavy alcohol users did show implicit negative associations toward alcohol (Wiers et al., 2002; De Houwer et al., 2004), suggesting that implicit substance-related negative associations may be common in heavy substance users regardless of the IAT type. We observed a similar pattern regarding the explicit negative expectancies as both groups showed significant explicit negative expectancies, but in heavy cannabis users they were less strong compared to controls. These findings imply that cannabis users generally have a less negative (but still negative) attitudes toward cannabis compared to the general population.

Interestingly, higher weekly cannabis use was associated with stronger implicit negative associations. This suggests that more heavy (problematic) users have implicit negative association toward cannabis, similarly to the controls. A similar relationship was observed regarding explicit negative expectancies: the higher cannabis related problems (CUDIT) the stronger the explicit negative expectancies toward cannabis. This finding is in accordance with studies showing that stronger negative expectancies were related to relapse in heavy drinkers (Jones and McMahon, 1994, 1996). In contrast to our hypothesis, we did not observe any other association between implicit or explicit associations toward cannabis and measures of cannabis use, cannabis use related problems, or craving. In a subsample of the heavy cannabis users we previously showed that the cannabis approach bias, as measured with a joystick approach avoidance task, could predict escalation of cannabis use 6 months later (Cousijn et al., 2011). A *post hoc* analysis with the participants included in the Cousijn et al. (2011) study indicated that implicit memory associations did not significantly predict future cannabis use (associations IAT measures with change in cannabis use and cannabis use related problems over 6 months was *r*^2^ < 0.078, *p* > 0.11). All together, these findings suggest that, in contrast to other substances of abuse like alcohol and nicotine (Wiers et al., 2007a), the relationship between implicit affective associations and cannabis abuse appears rather weak. Implicit associations toward cannabis may only play an important role in earlier stages of cannabis use, stimulating onset and repeated cannabis use rather than chronic cannabis use. This should then result in strong associations between implicit associations and cannabis use in a less heavy or regular group of cannabis users. Indeed a strong relationship between implicit associations and cannabis use has been found in sporadic/regular adolescent cannabis users (Ames et al., 2007). However, future research including different groups of cannabis users with varying levels of cannabis use is needed to clarify these issues.

Implicit and explicit positive-arousal and relaxed associations were significantly related within the control group only, suggesting that these implicit and explicit measures represent different motivational processes in cannabis users specifically. Negative implicit associations and explicit expectancies did not significantly correlate in both groups, indicating that implicit negative associations do not correspond with the self-reported negative expectancies of the participants. Moreover, similarly to earlier studies in heavy alcohol users (for an overview, see Wiers et al., 2007a), heavy cannabis users hold both implicit negative and positive-arousal associations toward cannabis, creating implicit ambivalence. The even stronger implicit negative associations with higher levels of cannabis use and explicit negative expectancies with higher levels of cannabis related problems (CUDIT-score) may then reflect an increasing conflict between the (craved) arousing effects of cannabis and the awareness of its negative consequences. However, future studies are needed to verify this.

For the current analyses, data from two separate studies were combined. Although implicit associations did not differ between studies, explicit positive-arousal expectancies toward cannabis were lower (but still positive) in individuals tested in the Academic Medical Center compared to those tested at the Psychology faculty of the University of Amsterdam. Experimental context may influence the retrieval of implicit and explicit associations from memory (Krank et al., 2005). The more formal hospital context could have specifically reduced positive-arousal expectancies toward cannabis. However, the difference in explicit positive-arousal expectancies could be due to other differences between the studies (i.e., experimenter, study design, specific cues).

In the Netherlands cannabis is decriminalized, which means that use and possession of cannabis are to a certain extent legal. Furthermore, cannabis can be purchased in so called “coffee-shops.” One might expect that this decriminalization would lead to less negative attitudes toward cannabis. However, in the present study both heavy cannabis users and controls had implicit and explicit negative associations toward cannabis, which is similar to results from studies conducted in countries with dissimilar cannabis policies or studies investigating legal substances of abuse like alcohol (Wiers et al., 2002; Field et al., 2004). This leads to the assumption that associations toward cannabis are not dependent on drug policy, which is in line with studies that failed to find that frequency and quantity or prevalence of cannabis use is influenced by drug policy (MacCoun and Reuter, 1997; Reinarman et al., 2004). For further research it would be interesting to test this hypothesis across countries.

Some limitations have to be taken into account. Among the heavy cannabis users more participants also smoked tobacco compared to controls. Since tobacco and cannabis cigarets show resemblance (Benett, 2008), it is possible that the implicit associations we observed toward cannabis partly reflect tobacco associations activated in tobacco smokers. However, IAT scores did not differ between smokers and non-smokers and scores for nicotine dependence did not significantly correlate with any of the IAT scores. Nevertheless, since almost all cannabis users smoked cannabis combined with tobacco, we cannot entirely discriminate between cannabis and tobacco effects. Concerning methodological issues, it has been argued that the IAT effect could be caused by salience of stimuli rather than by implicit attitudes, that is, the “figure ground effect” (Rothermund and Wentura, 2004). Although we cannot rule out the possibility of confounding figure ground effects, it has been shown that alcohol-arousal associations could not be explained by figure ground effects (Houben and Wiers, 2006). Finally, it has been suggested that extra-personal associations (i.e., cultural norms) could (at least in part) contaminate the IAT effect (Houben and Wiers, 2007). These extra-personal associations are suggested to be irrelevant to our personal behavior, which may explain why we observed only a weak association between implicit associations and measures of cannabis use and cannabis use related problems.

In summary, the present study demonstrates that both, controls and heavy cannabis users have implicit negative associations and explicit negative expectancies toward cannabis; however, these associations were stronger in controls. Moreover, heavy cannabis users showed stronger implicit arousal associations compared to controls. In contrast to other substances of abuse, implicit and explicit associations toward cannabis appear to be only weakly associated with cannabis use, suggesting only a limited role of implicit associations in cannabis abuse.

## Conflict of Interest Statement

The authors declare that the research was conducted in the absence of any commercial or financial relationships that could be construed as a potential conflict of interest.

## Footnotes

^1^All analyses were performed a second time omitting the variable “study.” Results and interpretations were similar.
